# Molecular surveillance of arboviruses circulation and co-infection during a large chikungunya virus outbreak in Thailand, October 2018 to February 2020

**DOI:** 10.1038/s41598-022-27028-7

**Published:** 2022-12-24

**Authors:** Sarawut Khongwichit, Watchaporn Chuchaona, Sompong Vongpunsawad, Yong Poovorawan

**Affiliations:** Center of Excellence in Clinical Virology, Faculty of Medicine, Chulalongkorn University, 1873 Rama 4 Road, Pathumwan, Bangkok, 10330 Thailand

**Keywords:** Infectious-disease diagnostics, Dengue virus, Viral epidemiology, Viral genetics

## Abstract

A large national outbreak of chikungunya virus (CHIKV) was recently reported in Thailand. While dengue virus (DENV) infection tends to occur year-round with an upsurge in the rainy season, Zika virus (ZIKV) also circulates in the country. The overlap in the distribution of these viruses increased the probability of co-infections during the heightened CHIKV activity. By examining 1806 patient serum samples submitted for CHIKV diagnostics from October 2018-February 2020 (511 CHIKV-negatives and 1295 CHIKV-positives), we used real-time reverse transcription-polymerase chain reaction to identify DENV and ZIKV individually. A total of 29 ZIKV and 36 DENV single-infections were identified. Interestingly, 13 co-infection cases were observed, of which 8 were CHIKV/DENV, 3 were CHIKV/ZIKV, and 2 were DENV/ZIKV. There were six DENV genotypes (13 DENV-1 genotype I, 10 DENV-2 Asian I, 10 DENV-2 Cosmopolitan, 6 DENV-3 genotype I, 2 DENV-3 genotype III, and 5 DENV-4 genotype I). Additionally, ZIKV strains identified in this study either clustered with strains previously circulating in Thailand and Singapore, or with strains previously reported in China, French Polynesia, and the Americas. Our findings reveal the co-infection and genetic diversity patterns of mosquito-borne viruses circulating in Thailand.

## Introduction

Important mosquito-borne viruses that cause human illness in Thailand include dengue virus (DENV), chikungunya virus (CHIKV), and Zika virus (ZIKV). Most people infected with DENV, ZIKV, or CHIKV share common symptoms, including high fever, rash, muscle pain, and joint pain^1^. Dengue is the highly prevalent mosquito-borne viral disease transmitted primarily by *Aedes spp.* of mosquito, which also serve as vectors for CHIKV and ZIKV. DENV exhibits hyper-endemic transmission in rural and urban areas of Thailand, with an average of 50,000 cases per year^2^. The average dengue incidence rate in Thailand between 2018 and 2020 was 144 per 100,000 population, with 295 deaths^3^. Four antigenically distinct serotypes of DENV have been reported (DENV-1, DENV-2, DENV-3, and DENV-4), and these serotypes exhibit 60–70% genome sequence similarity^4^. Each DENV serotype is further categorized into distinct genotypes depending on genetic divergence. Although all four DENV serotypes are generally found in dengue-endemic areas worldwide, genotypes within each virus serotype usually circulate in different geographic regions^5^. Both genotype replacement and co-circulation of multiple genotypes have been linked with virulence^6,7^.

ZIKV is closely related to DENV, as both are members of the genus *Flavivirus*. ZIKV has probably circulated in Thailand at a low but sustained level since at least 2002^8^. It is possible that ZIKV infection is commonly misdiagnosed as dengue fever or another illness due to the lack of a specific assay for ZIKV infection. However, a surge in the number of reported Zika fever cases in Thailand occurred in 2016–2017, with more than 1500 confirmed ZIKV infections^9^. It is plausible that this signaled the beginning of a large wave of ZIKV infections. After 2016–2017, Zika cases continued to be reported in Thailand, with 690, 142, and 144 confirmed cases in 2018, 2019, and 2020, respectively^10^. From 2016 to 2020, the Bureau of Epidemiology, Ministry of Public Health of Thailand reported 213 cases of pregnant women with confirmed ZIKV infection, including 11 women who had miscarriages, four of which were confirmed to be associated with ZIKV infection.

CHIKV is a mosquito-borne virus of the family *Togaviridae*, genus *Alphavirus*. CHIKV causes chikungunya fever (CHIKF). The first significant documented urban outbreak of chikungunya in Bangkok, Thailand, occurred in the 1960s^11^. From 2008 to 2009, a large CHIKV outbreak in Thailand affected mainly the southern region of Thailand, was driven by an albopictus-adaptive E1-A226V mutation in the East/Central/South African (ECSA) genotype, leading to approximately 54,000 infections. Ten years later, CHIKV infection re-emerged in Thailand, with > 27,000 cases reported by the end of 2020^12^. Our previous study found that the re-emergence of CHIKV outbreaks in Thailand was sustained by circulation of strains harboring two mutations, E1-K211E and E2-V264A, in the E1-226A background^13^. These mutations were correlated with increased infectivity, dissemination, and transmission in *Ae. aegypti*^14^. Of a total of 1,806 samples from our previous cohort study, 1295 were confirmed as CHIKV-positive and 511 as CHIKV-negative.

In Thailand, CHIKV, DENV, and ZIKV co-circulate, and the symptoms of infection with these mosquito-borne viruses can be indistinguishable, resulting in underdiagnosis of specific infections^1,15^. Surveillance of mosquito-borne viruses in endemic areas is necessary for enhance monitoring disease trends and identifying outbreaks. Therefore, this study aimed to investigate the prevalence of DENV, ZIKA and co-infection with DENV/ZIKV/CHIKV from the archived samples of a chikungunya cohort study of chikungunya suspected patients during the massive outbreak of CHIKV in Thailand from October 2018 to February 2020. We also characterized the genetic diversity of the detected DENV and ZIKV.

## Results

### Demographic and clinical presentation of DENV and ZIKV infection

ZIKV and DENV were initially screened using one-step real-time RT-PCR analysis of serum from the 511 CHIKV-negative patients. Of the 511 serum samples tested (Table 1) (Supplementary Table 1), 5.7% (29/511) were positive for ZIKV mono-infection, and 7.0% (36/511) were positive for DENV mono-infection. Two samples were positive for both ZIKA and DENV. Serotype-specific multiplex RT-PCR was subsequently performed to identify the DENV serotype of each positive sample. Of the 38 dengue infection cases, DENV-1 was detected in 11 (28.9%), DENV-2 in 17 (44.7%), DENV-3 in 5 (13.2%), and DENV-4 in 5 (13.2%). Study participants infected with ZIKV and DENV were further categorized according to sex, age, and clinical symptoms. Of those infected with ZIKV, 51.7% were male, and the remaining were female. The prevalence was highest among participants 31–40 years of age (34.5%), followed by those aged 41–50 (20.7%). Rash was significantly associated with ZIKV virus infection, with 82.8% of patients presenting with this symptom (*p* < 0.001), followed by fever (79.3%). Arthralgia and conjunctivitis of ZIKV mono-infection cases were found in 51.7 and 17.2%, respectively, were not significantly associated with the disease. Here, no neurological involvement was observed in any of the ZIKV-infected cases. Of those infected with DENV, 52.8% were male, while 47.2% were female. Gender was not statistically significant in both ZIKV and DENV mono-infection cases. The majority of DENV-confirmed cases were found in participants aged 21–30 (47.2%), followed by those aged 31–40 years (19.4%) and 41–50 years (13.9%), while the lowest prevalence was observed in patients aged 11–20 (2.6%). In this study, all DENV-infected cases represent fever. Arthralgia was reported in 52.8% of DENV-confirmed cases (*p* < 0.001). Rash was observed in 27.8% of DENV cases with no significant predictor of disease.

**Table 1:** Demographic and clinical presentation of ZIKV and DENV cases confirmed by RT-PCR screening of CHIKV-negative sample (n = 511).

Variable	Total cases (n) CHIKV negative	ZIKV Mono-infection n (%)	DENV Mono-infection n (%)	DENV/ ZIKV co-infection n (%)	DENV-1 n (%)	DENV-2 n (%)	DENV-3 n (%)	DENV-4 n (%)	Undifferentiated fever, n (%)
**Gender**									
Male	241	15 (51.7)	19 (52.8)	1 (50)	7 (35.0)	6 (30.0)	3 (15)	4 (20)	206 (46.4)
Female	270	14 (48.3)	17 (47.2)	1 (50)	4 (22.2)	11 (61.1)	2 (11.1)	1 (5.6)	238 (53.6)
Total	511	29 (5.7)	36 (7.0)	2 (100)	11 (28.9)	17 (44.7)	5* (13.2)	5* (13.2)	444 (86.9)
**Age (years)**									
≤ 10	45	1 (3.4)	3 (8.3)	–	1 (9.1)	2 (11.8)	–	–	41 (9.2)
11–20	55	4 (13.8)	1 (2.8)	–	1 (9.1)	–	–	–	50 (11.3)
21–30	118	3 (10.3)	17 (47.2)	–	3 (27.3)	11 (64.7)	–	3 (60)	98 (22.1)
31–40	115	10 (34.5)	7 (19.4)	1 (50)	3 (27.3)	2 (11.8)	1 (20)	2 (40)	97 (21.8)
41–50	85	6 (20.7)	5 (13.9)	–	1 (9.1)	1 (5.9)	3 (60)	–	74 (16.7)
> 50	93	5 (17.2)	3 (8.3)	1 (50)	2 (18.2)	1 (5.9)	1 (20)	–	84 (18.9)
**Symptom**									
Fever	484	23 (79.3)	36 (100)	2 (50)	11 (100)	17 (100)	5 (100)	5 (100)	423 (95.3)
Rash	154	24 (82.8)	10 (27.8)	2 (50)	4 (36.4)	5 (29.4)	1 (20)	2 (18.2)	118 (26.6)
Arthralgia	226	15 (51.7)	19 (52.8)	–	6 (54.5)	6 (35.3)	4 (80)	3 (27.3)	192 (43.2)
Conjunctivitis	31	5 (17.2)	–	–	–	–	–	–	26 (5.6)

*One case involved DENV and ZIKV co-infection.

### DENV/ZIKV/CHIKV co-infection

**Figure 1:**
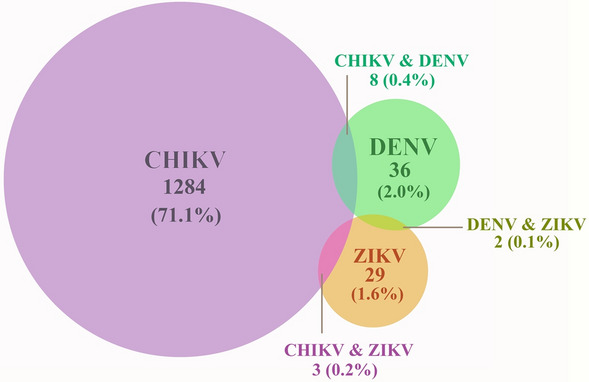
Venn diagram of DENV, ZIKV, and CHIKV mono-infection and co-infections found in studied samples. 1806 serum (511 CHIKV-negative and 1295 CHIKV-positive patients) from our previous chikungunya cohort study^13^ were retrospectively detected for ZIKV and DENV by using RT-PCR. A total of 29 ZIKV mono-infection cases and 36 DENV mono-infection cases were identified. 13 co-infection cases were observed, of which 8 cases were DENV/CHIKV co-infection, 3 cases were ZIKV/CHIKV co-infection with ZIKV, and 2 cases were DENV/ZIKV co-infection.

As shown in Table 2 and Fig. 1, co-infection with DENV and ZIKV was observed in two cases. The serotype of DENV in the co-infection cases was DENV-3 in one case and DENV-4 in one case. Samples were also analyzed for possible co-infection involving CHIKV and two arboviruses, including ZIKV and DENV. Samples from 1295 CHIKF-confirmed cases were examined by one-step RT-PCR to detect ZIKV and DENV. A total of 0.8% (11/1,295) of the samples were positive for co-infection, of which 27.3% (3/11) were positive for ZIKV and 72.7% (8/11) positive for DENV. Among the CHIKV and DENV co-infection cases, 3 dengue serotypes were found, including DENV-1 in 2 cases, DENV-2 in 3 cases, and DENV-3 in 3 cases. The 13 acute fever cases exhibiting any co-infection had a fever for 1 to 4 days before serum was collected. As shown in Supplementary Table 1, the mean ± SD days onset of symptoms before the sample was collected was 2.3 ± 1.3, 3 ± 1.2, and 3 ± 2.1 in CHIKV/DENV, CHIKV/ZIKV, and DENV/ZIKV co-infection cases, respectively. For the mono-infection case, there were 6 ± 11.4 days in CHIKV mono-infection, 3 ± 1.7 days in DENV mono-infection, and 3 ± 1.5 days in ZIKV mono-infection cases. The highest mean age was observed in CHIKV/ZIKV co-infection cases (53 ± 7 years), while the mean age of DENV/ZIKV was 46 ± 15.6, CHIKV/DENV was 31.4 ± 12.0, and all of the single virus infected cases were lower than 40. Gender was not significantly associated with an increased prevalence of co-infection. All ZIKV co-infected patients had a rash, whereas rash was observed in only 2 of 8 patients with DENV and CHIKV co-infection. Joint pain was observed in 7 of 11 patients with CHIKV co-infection. Notably, there was no evidence of neurologic complications associated with ZIKV co-infection. In our study, dual infection of DENV/ZIKV/CHIKV was not associated with more severe clinical disease than a single infection.

**Table 2:** Demographic data, laboratory diagnostic test results, and clinical presentation of CHIKV/ZIKV/DENV co-infected cases.

Case number	1	2	3	4	5	6	7	8	9	10	11	12	13
Patient ID	BK 1702	BK 2004	BK 1610	BK 1824	BK 2261	BK 1667	BK 2408	BK 2589	BK 1812	BK 2351	BK 2699	BK 1763	BK 2742
Age (years)	59	28	43	37	40	12	36	34	50	61	48	35	57
Gender	M	F	F	F	F	M	F	M	M	F	M	F	M
Location	BKK	BKK	BKK	BKK	BKK	BKK	BKK	BKK	BKK	BKK	BKK	SKN	BKK
Day(s) of illness onset	2	2	5	1	1	2	3	2	2	4	4	1	4
Laboratory CHIKV diagnosis*													
CHIKV RT-PCR	+	+	+	+	+	+	+	+	+	+	−	−	−
CHIKV IgM	−	−	−	−	−	−	−	−	−	−	+	−	−
CHIKV IgG	−	−	−	−	−	−	−	−	−	−	+	−	−
CHIKV genotype*	E	E	E	E	E	E	E	E	E	E	ND	ND	ND
DENV RT-PCR	+	+	+	+	+	+	+	+	−	−	−	+	+
DENV serotype	D1	D1	D2	D2	D2	D3	D3	D3	−	−	−	D4	D3
DENV genotype	I	I	Cos	Cos	Cos	I	III	I	ND	ND	ND	I	I
ZIKV RT-PCR	−	−	−	−	−	−	−	−	+	+	+	+	+
ZIKV genotype	ND	ND	ND	ND	ND	ND	ND	ND	A	A	A	A	A
**Clinical presentation**													
Fever	Yes	Yes	Yes	Yes	Yes	Yes	Yes	Yes	Yes	Yes	Yes	Yes	Yes
Rash	Yes	No	Yes	No	No	No	No	No	Yes	Yes	Yes	Yes	Yes
Arthralgia	No	Yes	Yes	Yes	Yes	No	Yes	No	Yes	Yes	No	No	No
Conjunctivitis	No	No	No	No	No	No	No	No	No	Yes	No	No	No

*M* Male, *F* Female, *BKK* Bangkok, *SKN* Samut Sakhon, *E* East/Central/South African genotype, *D1* Dengue virus serotype 1, *D2* Dengue virus serotype 2, *D3* Dengue virus serotype 3, *D4* Dengue virus serotype 4, *I* Genotype I, *Cos* Cosmopolitan genotype, *III* Genotype III, *A* Asian genotype, + Positive result, − Negative result, ND Not done.

*Results from our previous cohort chikungunya study.

Case numbers 1–8 involved CHIKV/DENV co-infection, Case numbers 9–11 involved CHIKV/ZIKV co-infection, and Case numbers 12–13 involved DENV/ZIKV co-infection.

### Genotypic characterization of ZIKV detected in Thailand in 2019–2020

**Figure 2:**
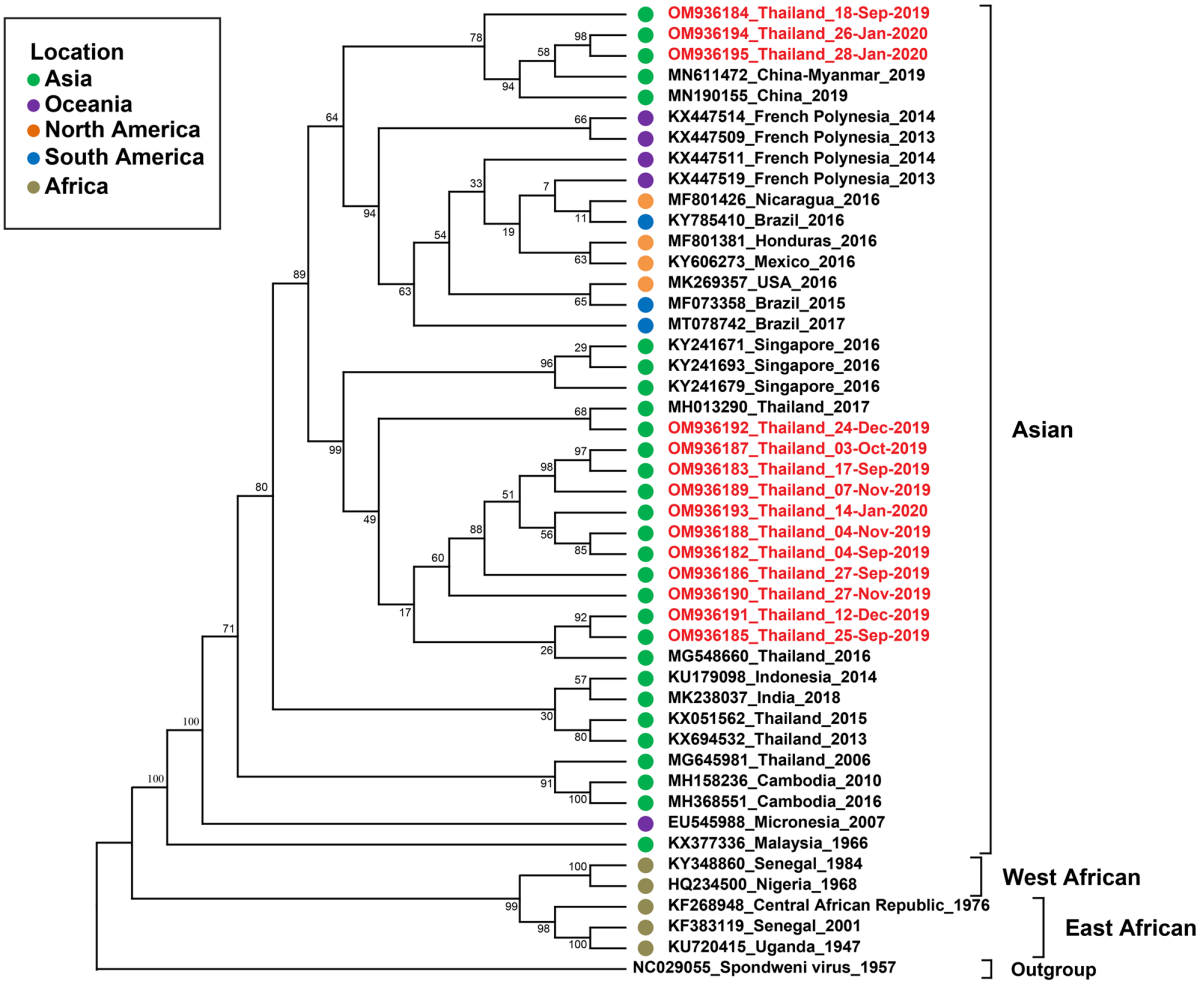
Phylogenetic analysis of the ZIKV structural protein gene coding region (C-prM-E). The maximum-likelihood tree of the ZIKV structural protein gene (C-prM-E) sequences was generated using the TNe + I + G4 model with 1000 ultrafast bootstrap replicates. Bootstrap values are indicated at branch nodes. ZIKV Thai strains from this study are shown in red.

**Figure 3:**
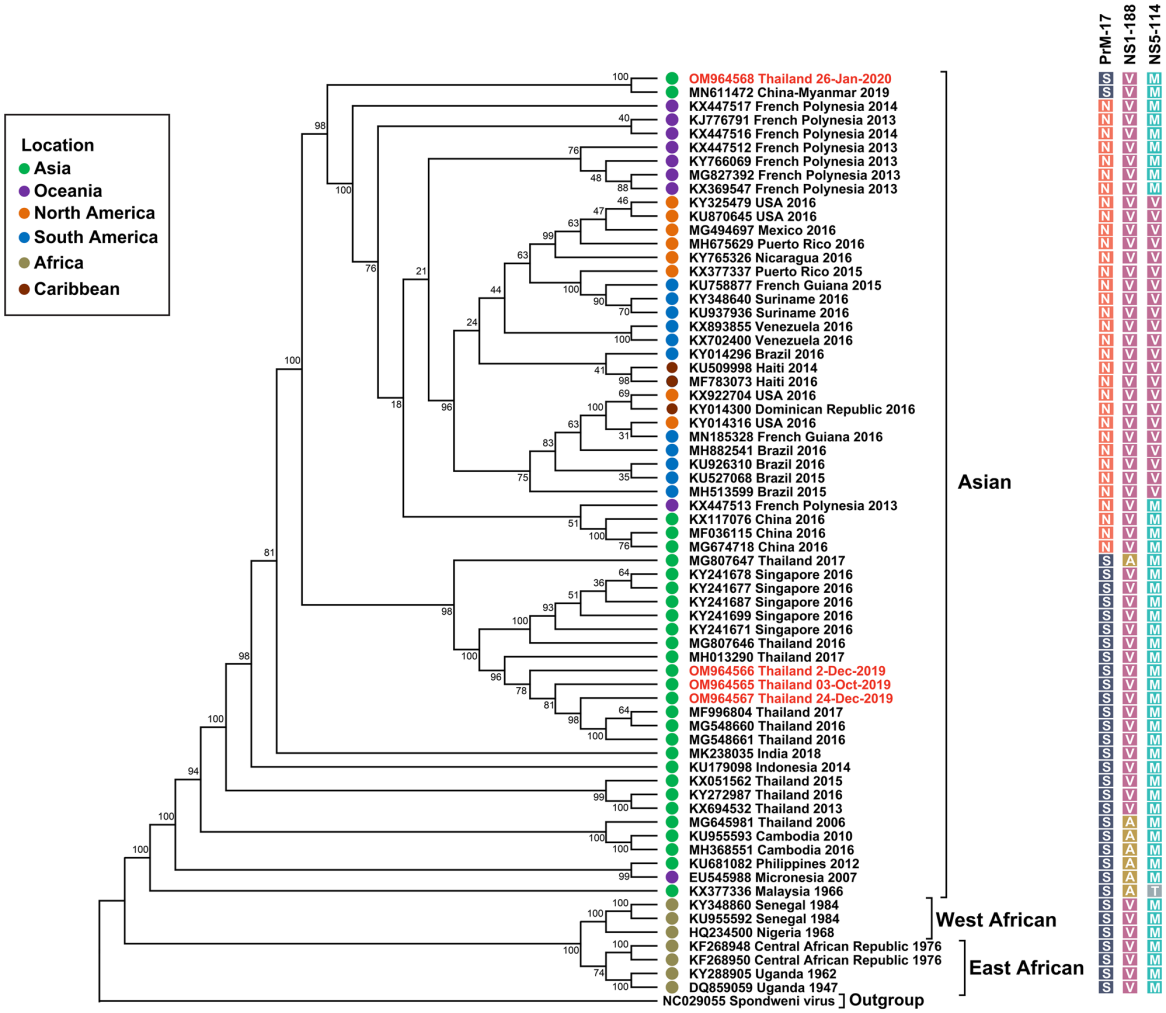
Phylogenetic analysis of the ZIKV complete coding sequence. The maximum-likelihood tree of ZIKV complete coding sequences was generated using the GTR + F + I + G4 model with 1000 ultrafast bootstrap replicates. Bootstrap values are indicated at branch nodes. ZIKV Thai strains from this study are shown in red. Different-colored squares show specific amino acid substitutions in prM, NS1, and NS5.

In this study, 34 acute febrile illness cases were confirmed by RT-PCR to have ZIKV infection (Supplementary Table 2). A maximum-likelihood phylogenetic tree of ZIKV was constructed based on the structural protein-coding region (C-prM-E) of ZIKV isolates from 14 patients with the other ZIKV sequences available from the GenBank database. Moreover, a phylogenetic tree based on the complete coding sequence was also constructed. The phylogenetic analysis indicated that all ZIKV isolates identified from laboratory-confirmed cases during 2019–2020 belonged to the Asian lineage (Figs. 2 and 3). However, the ZIKV Thai strain of 2019–2020 formed two main clusters. Most of the ZIKV Thai strains formed a cluster with ZIKV strains identified in earlier epidemiologic profiling studies in Thailand and Singapore. The other cluster consisted of strains closely related to viruses identified in China, French Polynesia, and various locations in the Americas. We then examined the degree of nucleotide sequence similarity among our Thailand isolates from the two different clusters, previous Thai strains from 2016–2017, and strains from different geographic regions by pairwise alignment of the complete coding sequences. The complete coding sequence nucleotide similarity among Thai strains in this research (2019–2020) ranged between 98.47 and 99.24%. As described above, our ZIKV Thai strains isolated in 2019–2020 formed two clusters. ZIKV Thai strains of 2019 (OM964565–OM964567) of the first cluster shared high nucleotide sequence similarity with previous Thai stains isolated in 2016–2017 (99.26–99.60%) and ZIKV strains isolated in Singapore in 2016 (99.29–99.42%).

In contrast, the ZIKV Thai strain of 2020 (OM964568) from the distinct cluster shared the highest nucleotide similarity (99.64%) with a strain isolated from a Chinese tourist returning from Myanmar in 2019. The complete coding sequence of the Thai strain of 2020 (OM964568) also showed high nucleotide identity with sequences of ZIKV strains isolated in French Polynesia in 2013–2014 (99.07–99.16%) and strains reported previously in the Americas (98.77–99.07%), higher than the identity of previous Thai strains isolated between 2006 and 2017 (98.11–98.76%). According to a previous report^16^, strains of the Asian lineage can be classified into four main genotypes (SAM, SVM, NVM, and NVV) based on differences in amino acids in three proteins, residue 17 in the prM protein, residue 188 in the NS1 protein, and residue 114 in the NS5 protein. Thus, the specific amino acid patterns in these three proteins of ZIKV Thai strains of 2019–2020 were then analyzed. Our ZIKV Thai strains of 2019–2020 were identified as SVM genotype, similar to previous Thai isolates and other Southeast Asian isolates. Some of the ZIKA Southeast Asian isolates and the Micronesian strain of 2007 were SAM genotype. By contrast, the ZIKV French Polynesian strains of 2013–2014 were NVM genotype, and all ZIKV isolates from the Americas contained the NS5-114 V sequence and were therefore classified as NVV genotype (Fig. 3).

### Genotypic characterization of DENV detected in Thailand in 2019–2020

Based on analyses of 1806 patient serum samples obtained during our chikungunya cohort study from October 2018 to February 2020, 46 patients were confirmed by RT-PCR to have DENV infection (Supplementary Table 3). Of the 46 isolates, 13 were DENV-1, 20 DENV-2, 8 DENV-3, and 5 were DENV-4. The E protein gene region of DENV-1, DENV-2, and DENV-4 and the C-prM region of DENV-3 were amplified for phylogenetic analysis to enhance understanding of the epidemiologic and molecular characteristics of DENV isolates in Thailand during the period 2018–2020.

**Figure 4:**
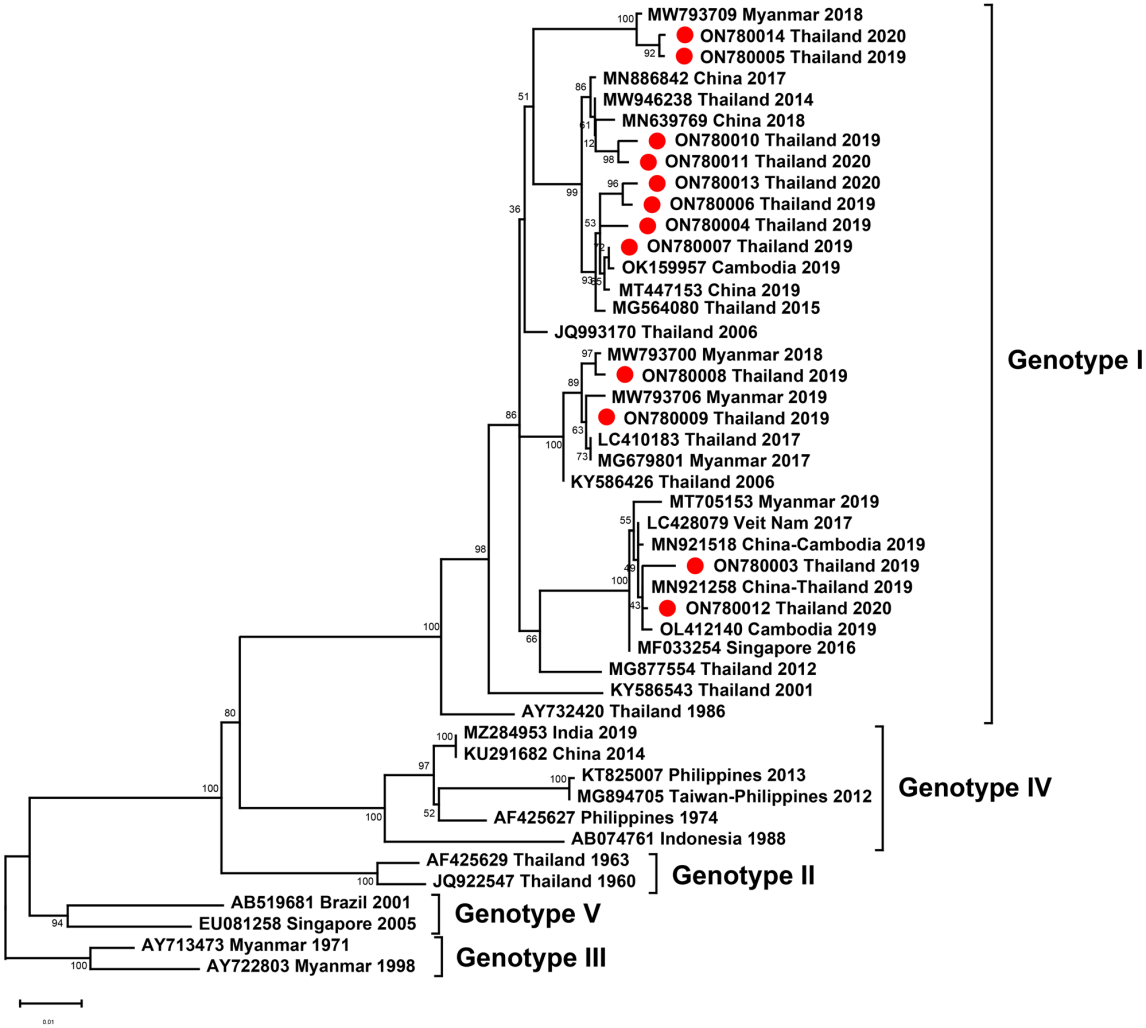
Phylogenetic analysis of DENV-1. The maximum-likelihood tree of DENV-1 was constructed from partial E gene sequences (1362 nucleotides) of DENV-1 Thai strains isolated in this study and strains selected from the GenBank database. The tree was generated using the TIM2 + F + G4 model with 1000 ultrafast bootstrap replicates. Bootstrap values are indicated at branch nodes. Red dots denote DENV Thai strains from this study. Black brackets on the right indicate DENV-1 genotype.

All DENV-1 Thai isolates from 2019–2020 belonged to genotype I, similar to the contemporary isolates from other countries in Southeast Asia, China, and most previously circulating strains in Thailand. Notably, the phylogenetic tree also showed that our DENV-1 Thai strains did not cluster in the same clade, indicating the presence of intra-genotype variability among genotype I of DENV-1 strains circulating in Thailand (Fig. 4).

**Figure 5:**
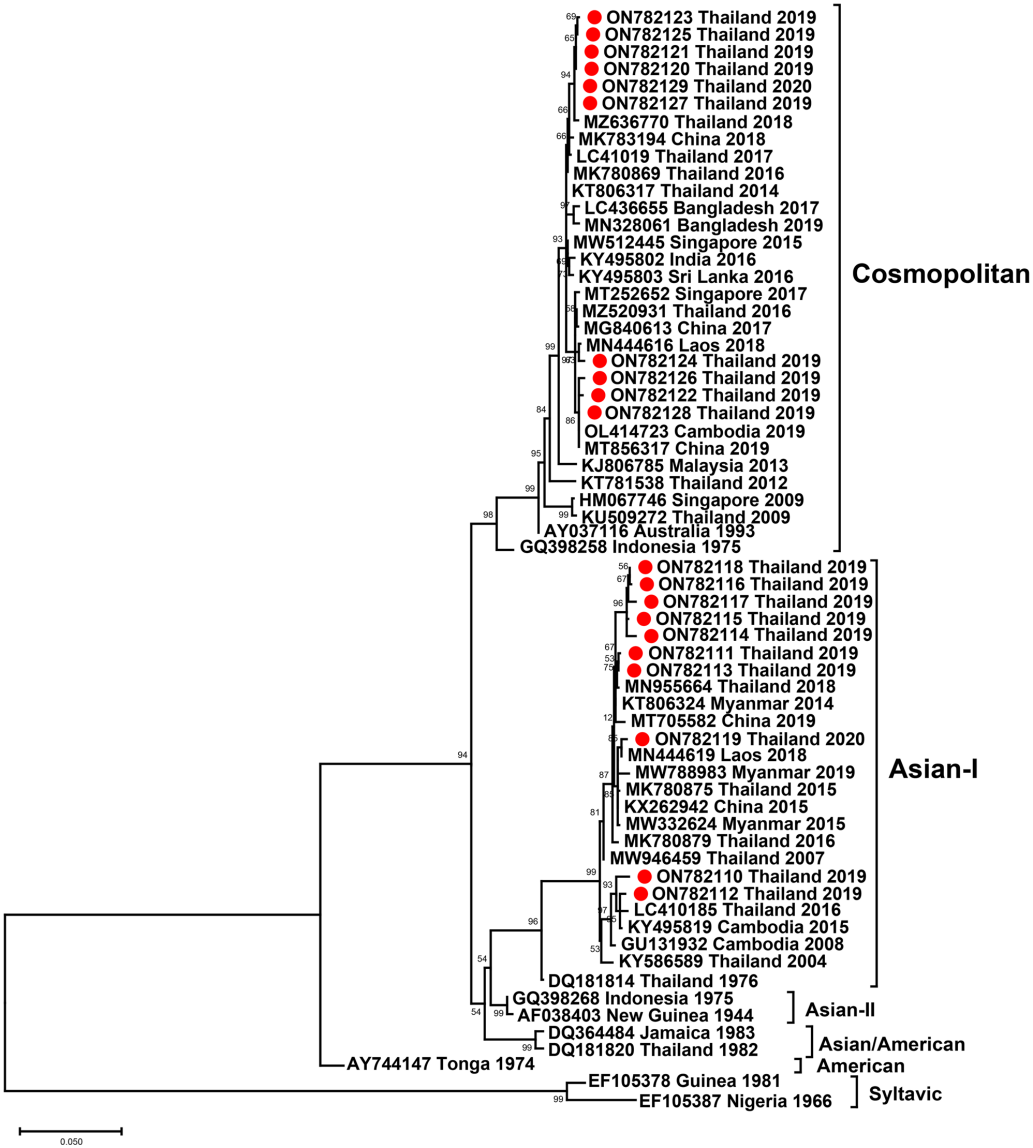
Phylogenetic analysis of DENV-2. The maximum-likelihood tree of DENV-2 was constructed from partial E gene sequences (1090 nucleotides) of DENV-2 Thai strains isolated in this study and strains selected from the GenBank database. The tree was generated using the TIM2 + F + G4 model with 1000 ultrafast bootstrap replicates. Bootstrap values are indicated at branch nodes. Red dots denote DENV Thai strains from this study. Black brackets on the right indicate DENV-2 genotype.

For DENV-2, the phylogenetic tree showed that two different genotypes co-circulated in Thailand in 2019–2020. Ten isolates were identified as Asian I. Seven of the Asian I strains were closely related to one strain isolated in Thailand in 2018 and China in 2019. One strain was closely related to DENV-2, which was isolated in Laos in 2018, whereas the other two Asian I strains were related to the DENV-2 strain isolated in Cambodia and Thailand in 2015–2016. By contrast, the remaining 10 isolates were classified as the Cosmopolitan genotype. These isolates formed two different clades. Six Cosmopolitan strains clustered with previous Thai strains isolated in 2017–2018 and DENV-2 isolated in China in 2018, whereas the remaining Cosmopolitan Thai strains of 2019 were closely related to DENV-2 isolated from Cambodia in 2019, China in 2019, and Laos in 2018 (Fig. 5).

**Figure 6:**
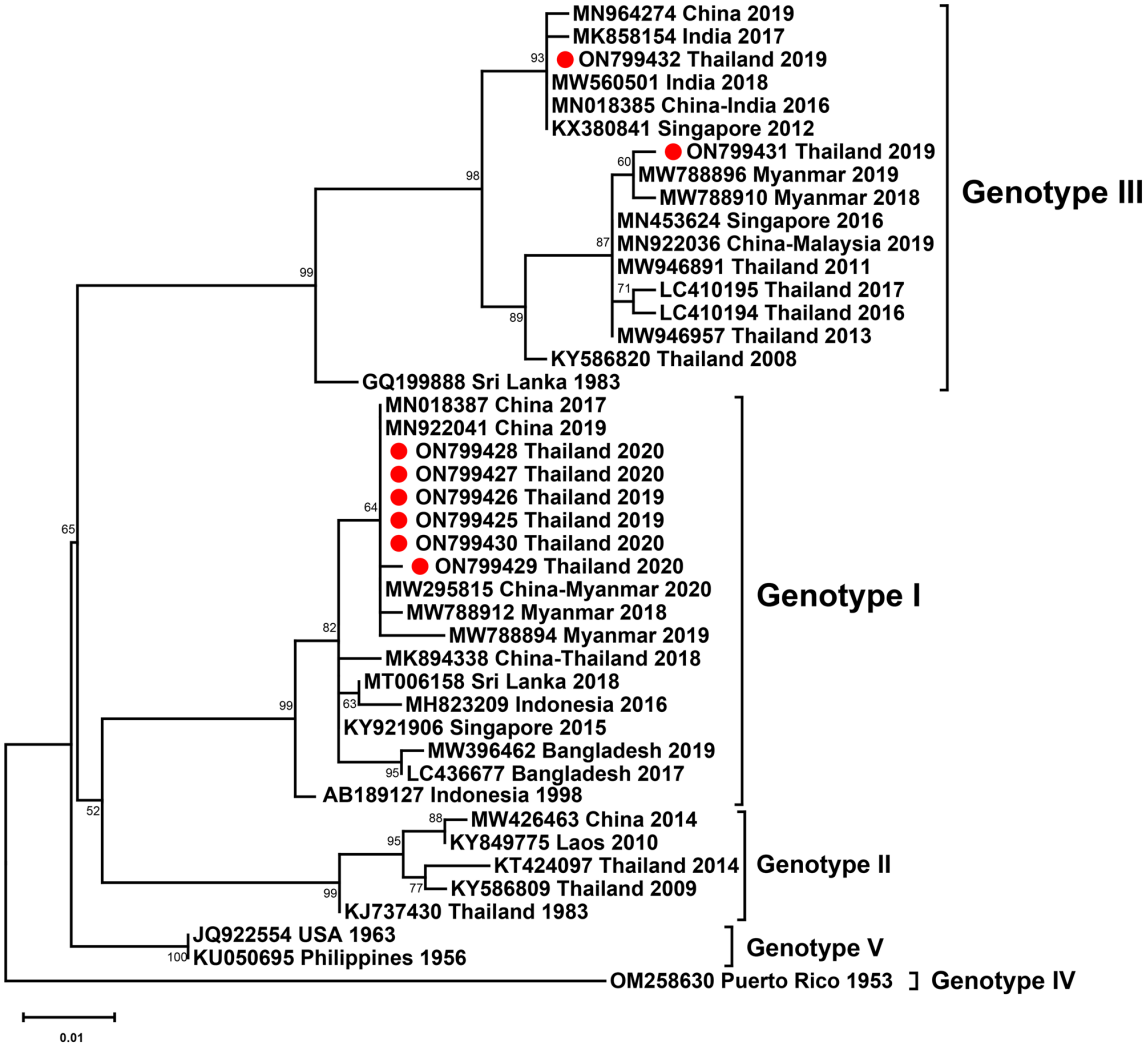
Phylogenetic analysis of DENV-3. The maximum-likelihood tree of DENV-3 was constructed from prM gene sequences (444 nucleotides) of DENV-3 Thai strains isolated in this study and strains selected from the GenBank database. The tree was generated using the TIM2e + I model with 1000 ultrafast bootstrap replicates. Bootstrap values are indicated at branch nodes. Red dots denote DENV Thai strains from this study. Black brackets on the right indicate DENV-3 genotype.

Of eight DENV-3–positive samples, eight partial C-prM sequences were examined, including four from DENV-3 isolates from 2019 and four from 2020. There were six DENV-3 genotype I isolates and two DENV-3 genotype III isolates, suggesting co-circulation of the two DENV-3 genotypes in Thailand between 2019 and 2020. Our DENV-3 genotype I strain of 2019–2020 clustered with strains from China and Myanmar collected between 2017 and 2019. One of the DENV-3 genotype III isolates clustered with Myanmar strains, whereas the other clustered with strains from India collected in 2017–2018, China in 2019, and from a Chinese traveler returning from India in 2016 (Fig. 6).

**Figure 7:**
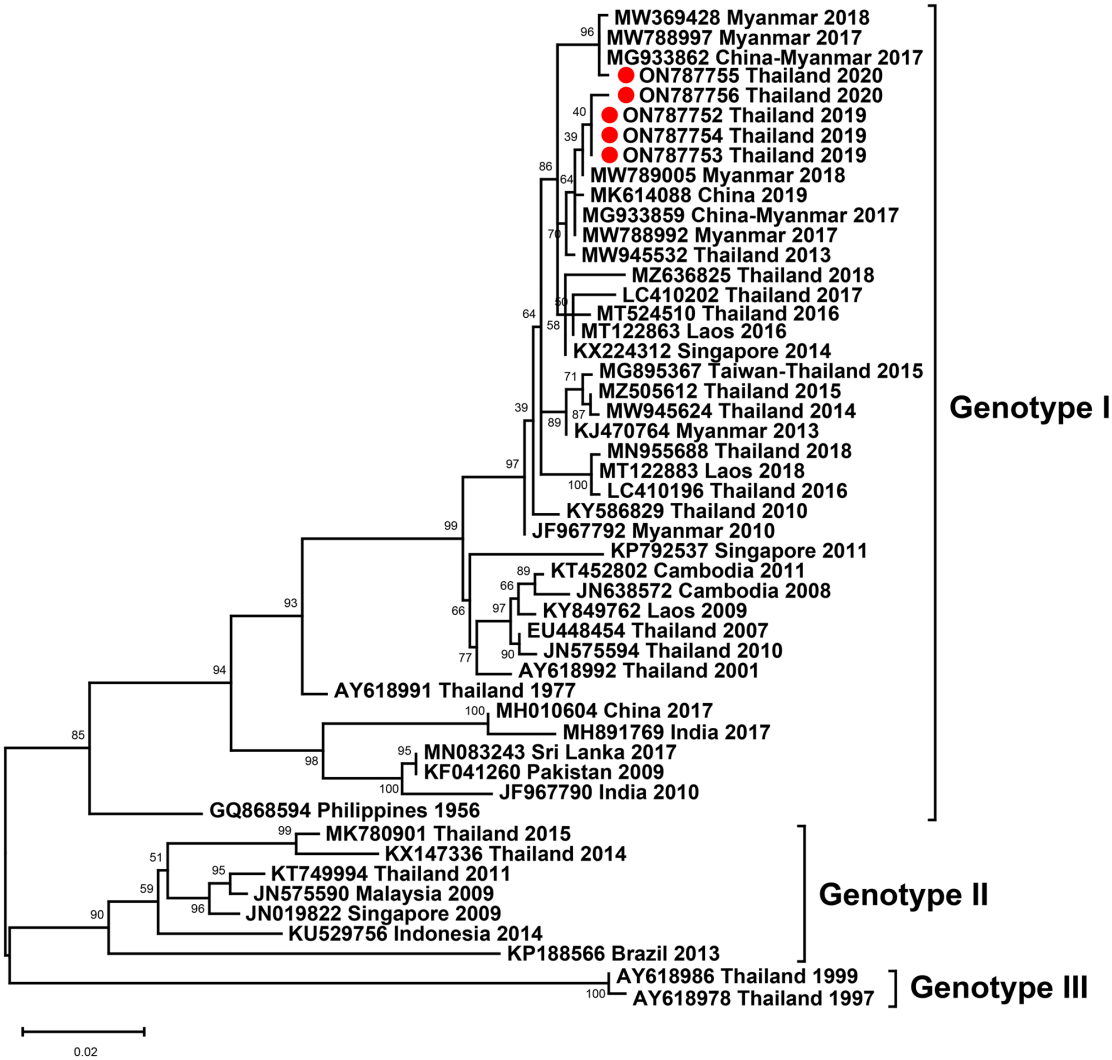
Phylogenetic analysis of DENV-4. The maximum-likelihood tree of DENV-4 was constructed from partial E gene sequences (724 nucleotides) of DENV-4 Thai strains isolated in this study and strains selected from the GenBank database. The tree was generated using the TIM + F + G4 model with 1000 ultrafast bootstrap replicates. Bootstrap values are indicated at branch nodes. Red dots denote DENV Thai strains from this study. Black brackets on the right indicate DENV-4 genotype.

Among five DENV-4–positive samples, a partial sequence of the DENV E gene of each sample was amplified and used to generate a phylogenetic tree with other strains from the NCBI database. Sequence analysis and the phylogenetic tree revealed that all DENV-4 Thai strains isolated in 2019–2020 belonged to genotype I and clustered with strains isolated in Myanmar in 2017–2018 (Fig. 7).

## Discussion

In this study, we took advantage of our previous chikungunya cohort study conducted from October 2018 to February 2020^13^ to examine evidence of ZIKV, DENV, and co-infection among these three pathogens using a nucleic acid amplification test. RT-PCR is the preferred method of diagnosis due to its high specificity, high sensitivity, and low cross-reactivity. We screened serum samples from our previous chikungunya cohort study collected during the large outbreak of CHIKV infection in Thailand for DENV and ZIKV. There were 511 CHIKV-negative serum samples and 1295 CHIKV-positive samples. A total of 36 patients were found to be mono-infected with DENV among the 511 patients with a CHIKV-negative test result. Similarly, other studies also identified DENV as a significant virus in patients with undifferentiated fever in Thailand^17^. Between October 2018 and February 2020, a total of 325 cases of Zika fever were reported in Thailand by the Bureau of Epidemiology, Ministry of Public Health^10^. Interestingly, we found 29 cases of ZIKV mono-infection, representing approximately 10% of the Zika fever cases reported by the Bureau of Epidemiology during the same period. These cases suggest the possibility of underdetection and underreporting of mosquito-borne virus infections in Thailand. Our findings also confirm the high rate of DENV, ZIKV, and CHIKV co-circulation in Thailand.

The clinical manifestations of illnesses caused by mosquito-borne viruses overlap with those of other co-circulating viruses, particularly DENV, ZIKV, and CHIKV. Infection with CHIKV, DENV, and ZIKV may be asymptomatic, undifferentiated acute febrile illness and self-managed; hence, the actual numbers of infection cases are likely to go underreported^18–20^. Although skin rash is commonly observed in cases of DENV, ZIKV, and CHIKV infection, we found that rash was more frequent in ZIKV-confirmed patients than in patients infected with DENV or CHIKV. Our finding was consistent with studies from Brazil^21^ and Mexico^22^. RT-PCR analyses indicated that DENV- and ZIKV-positive samples tended to have been collected early after onset of illness. The median time from onset of disease to DENV and ZIKV detection was 2 (range 1–9) days and 3 (range 1–7) days, respectively (Supplementary Tables 2 and 3). Other studies have reported that RT-PCR is highly sensitive for detecting these viruses in the early stages of infection^23,24^.

A total of 444 samples that were CHIKV negative by RT-PCR and IgM testing were also RT-PCR–negative for DENV and ZIKV. The timing of the test relative to disease stage is one factor that influences the accuracy of the diagnosis. The RT-PCR assay used here would have low sensitivity for serum samples collected late in the disease progression. Negative viral RNA detection results do not rule out a diagnosis of dengue or Zika fever because the viremic phase of DENV and ZIKV infection is short^25,26^. In the middle and latter stages of infection, serological tests are more effective than viral RNA detection. However, we did not perform serological testing or plaque reduction neutralization tests (PRNTs) due to the inadequate volume of serum samples. We also assumed that DENV- and ZIKV-negative test results could be due to fever caused by other pathogens. A recent acute undifferentiated fever etiology study in Thailand reported that in addition to DENV, acute undifferentiated fever can also occur with murine typhus, leptospirosis, influenza, and bacteremia^17^.

A further significant finding of this study was the detection of 13 cases of co-infection, which comprised eight cases of CHIKV/DENV co-infection, three cases of CHIKV/ZIKV co-infection, and two cases of DENV/ZIKV co-infection. Some studies have reported that co-infection with mosquito-borne viruses can exacerbate disease severity^27,28^. Mercado-Reyes et al. described seven fatalities related to DENV/CHIKV and CHIKV/ZIKV co-infection during epidemiologic monitoring of the ZIKV outbreak in Colombia between October 2015 and December 2016. Among the co-infection mortality cases, death was related to neurological manifestations, sepsis, and multiple organ failure in five cases, whereas the other two cases involved fetal death^28^. Nevertheless, of the co-infection cases in our study, fever was the most common clinical feature for all patients, and no severe symptoms were observed. Many previous studies have also reported that the clinical features of dual infections involving CHIKV/ZIKV^29^, CHIKV/DENV^30^, and DENV/ZIKV^31^ are not associated with worse outcomes and do not differ significantly in terms of clinical features compared with single infections with each virus. Moreover, one systematic review reported that only 9% of ZIKV–co-infected patients in cohort and cross-sectional investigations had complications^32^.

In addition to diagnostic testing, investigations of viral genetic variations are essential for infection prevention and understanding how a virus spreads is necessary for further development of antiviral agents. In this study, phylogenetic analyses demonstrated that in Thailand, ZIKV belongs to the Asian genotype but forms two main clades. Most isolates clustered with previous Thai and Singapore strains isolated in 2016–2017. Isolates in the other clade were closely related to a ZIKV strain isolated in a Chinese traveler returning from Myanmar in 2019 as well as strains reported previously in French Polynesia and the Americas. Our findings are similar to those of a previous study that classified ZIKV from mosquitoes in 2016 in Thailand into two clades closely related to the Thai strain of 2013–2017 and the other clade related to viruses identified in the Americas^33^. Based on differences in the amino acid residues in three proteins−position 139 (localized in residue 17 of prM protein), 982 (188 in the NS1 protein), and 2634 (114 in NS5 protein)−the Asian lineage ZIKV isolates have been categorized into four major genotypes, SAM, SVM, NVM, and NVV^16^. Changes in a single amino acid residue at positions 139 and 982 are reportedly associated with more-severe disease and enhanced viral transmission^34,35^.

ZIKV exhibits a serine to asparagine substitution at position 139 (S139N) in prM, which arose during the ZIKV outbreak that occurred in early 2013 in French Polynesia before spreading throughout the Americas^36^. Yuan et al.^34^ reported that the S139N substitution in prM enhances ZIKV replication in human and mouse neural progenitor cells, resulting in more-severe microcephaly in infected mouse fetuses. The A982V substitution in the NS1 protein increases ZIKV NS1 secretion into the host circulation and substantially enhances transmission of ZIKV in mosquito vectors^35^. Additionally, the NS1 A982V mutation enhances ZIKV replication via inhibition of interferon-β induction^37^. A change of methionine or threonine to valine at position 2634 (M//T2634V) from the start codon of the genome or residue 114 in the NS5 protein is a unique molecular hallmark of all American strains isolated in 2015–2016^36,38^.

In contrast, strains from Southeast Asia, the Pacific, and Africa harbor 2634M, and a ZIKV Malaysian strain isolated in 1966 harbored 2634T^36^. It could be hypothesized that the M2634V substitution in the American strains is responsible for the increased outbreak potential of the mutant virus in the Americas. However, recent studies have shown that the insignificant M2634V mutation in the NS5 protein enhances viral replication and transmission potential, indicating that the M2634V substitution is not responsible for enhancing the ZIKV epidemic potential^39,40^. Here, we found that the ZIKV Thai strains isolated in 2019–2020 were SVM, similar to most previously isolated Thai and other Southeast Asian strains. Although the SVM strains do not carry the S139N mutation, which is associated with increased neurovirulence of ZIKV^34^, it was reported that infection with SVM ZIKV variants can lead to congenital Zika syndrome and microcephaly. However, one crucial factor in the development of microcephaly in a fetus or infant is the timing of ZIKV infection during pregnancy. A high risk of microcephaly with ZIKV infection is observed in the first trimester^41^.

Among DENV-positive samples, all four serotypes were detected in this study, indicating that all four DENV serotypes co-circulated in Thailand in 2019–2020. The most common serotype was DENV-2, consistent with a 2020 report from the Bureau of Vector-Borne Diseases, Department of Disease Control, Ministry of Public Health, Thailand. In this study, six different DENV genotypes were identified among the four serotypes. DENV-1 comprises genotypes I, II, III, IV, and V^42^. All DENV-1 isolates detected in this study belonged to genotype I, which was first documented in Thailand in 1981^43^. Since that time, DENV-1 genotype I has been routinely isolated in Thailand and several other countries in Southeast Asia^44,45^. Interestingly, our DENV-1 phylogenetic tree showed that previous and present genotype I Thai strains did not cluster in the same clade. Most of the Thai strains in each clade were closely related to genotype I strains from neighboring countries, demonstrating intra-genotypic variation of DENV-1 genotype I strains in Thailand and a genetic link between DENV-1 circulating in Thailand and other countries in Southeast Asia. We also observed geographic expansion of DENV-1 genotype I strains from Southeast Asia to East Asia as a result of the close relationship between Thai and Cambodian with DENV-1 isolated from China. A previous report also showed that an epidemic involving DENV-1 in China was related to infected Chinese tourists returning from Thailand^46^.

Six distinct genotypes of DENV-2 have been identified^47^. The sylvatic genotype circulates in West Africa and Southeast Asia and frequently exhibits enzootic cycles between forest mosquitoes and nonhuman primates. The American genotype is present in South and Central America. Over the past three decades, the Asian/American genotype circulating in the Americas has caused several epidemics in South and Central America^48^. This genotype was previously the dominant circulating genotype in Southeast Asia, but Asian I genotype viruses began to replace the Asian/American genotype in 1992^49^. The Asian I genotype of DENV-2 is frequently detected in many Asian countries^50,51^. The Asian II genotype includes strains from Asia, among which the DENV-2 Cosmopolitan genotype is the most prevalent globally^52^. A phylogenetic analysis of DENV-2 isolates based on envelope region sequence indicated that both the Asian I and Cosmopolitan genotypes co-circulated in Thailand during 2019–2020, especially in Bangkok. Previous studies also found that both genotypes of DENV-2 co-circulated in Southern Thailand in 2015–2016^45^.

Five DENV-3 genotypes (I-V) have been identified to date^53^. DENV-3 genotype II was the predominant genotype in Thailand in the 1970s^54^. Displacement of genotype II by genotype III in Thailand was reported in 2008^55^. DENV-3 genotype I was first documented in 1988^56^. In 2015, DENV-3 genotype I re-emerged and co-circulated with DENV-3 genotype III in southern Thailand, but genotype III was detected more frequently than genotype I^45^. In contrast, other studies have reported that only genotype III circulated in Thailand in 2016–2017^57^. The present study found that DENV-3 genotypes I and III co-circulated in Bangkok in 2019–2020, with genotype I detected at higher frequency than genotype III. This evidence suggests there has been a genotypic shift in DENV-3 in Thailand. A study in Myanmar, a neighboring country of Thailand, also reported co-circulation of the emerging genotype I in that country from 2017 to 2019^58^. Our phylogenetic analysis showed that DENV-3 genotype I is closely related to DENV-3 strains from Myanmar, indicating the possibility of the virus spreading across countries. In addition, our DENV-3 genotype III formed two phylogenetic clusters, one closely related to strains from Myanmar and the other related to Indian strains, indicating the spread of the virus between countries in Southeast Asia and South Asia.

DENV-4 was rarely isolated in this study. Phylogenetic analysis revealed that the DENV-4 Thai strains isolated in 2019–2020 were predominantly of genotype I and closely related to the DENV-4 isolate from Myanmar and a Chinese traveler who visited Myanmar suggesting the spread of the virus to a nearby country and wider geographic expansion of the virus from Southeast Asia to East Asia.

In conclusion, we found that cases of dengue fever and Zika infection were underestimated and underreported during the large CHIKV outbreak that occurred in Thailand between October 2018 and February 2020. A possible reason for this underestimation and underreporting is that infections involving CHIKV, DENV, and ZIKV can cause similar but widely varying clinical presentations. As such, accurate laboratory diagnosis is imperative. We thus highlight the co-infection and co-circulation of these three mosquito-borne viruses in Thailand. ZIKV circulation in Thailand during 2019–2020 was the Asian genotype divided into two clades: one group related to previous circulating Thai and Singaporean strains, and the other closely related to viruses identified in China, French Polynesia, and the Americas. Four DENV serotypes were detected, including six genotypes consisting of 13 DENV-1 genotype I, 10 DENV-2 Asian I, 10 DENV-2 Cosmopolitan, 6 DENV-3 genotype I, 2 DENV3 genotype III, and 5 DENV-4 genotype I. Notably, most of the genotypes were also classified into multiple clades. Most confirmed cases occurred in Bangkok, the capital and most populous city in Thailand. In addition, molecular characterization of the viral genomes revealed the degree of genetic variation between ZIKV and the four DENV serotypes. Bangkok undoubtedly serves as the epicenter for mosquito-borne virus diversity in Thailand. Continuous monitoring of viral genetics, in conjunction with development of a surveillance database, may enhance our understanding of viral genotype changes and their association with epidemiology, thereby helping prevent or control outbreaks involving these viruses.

## Materials and methods

### Ethics statement

The study was carried out in compliance with the guidelines of the Institutional Review Board of the Ethics Committee of the Chulalongkorn University Faculty of Medicine, which evaluated and approved the study (IRB no. 710/64). The Institutional Review Board of the Ethics Committee for Human Research waived the need for informed consent from participants because all clinical specimens were de-identified. All experiments were performed following relevant guidelines and regulations.

### Sample collection

This work retrospectively investigated serum samples used in a previous study^13^. The present study examined 1806 serum samples from the previous cohort study collected from patients with suspected CHIKV infection from 13 provinces of Thailand between October 2018 and February 2020. A suspected case of chikungunya involves a patient with acute onset of fever, often reaching more than 38.5 °C, with or without arthralgia and rash, especially in a person who has resided or visited a known risk area of CHIKV transmission. The age of subjects varied from 3 to 97 years. In patients with suspected CHIKF, serum samples were collected and examined for the presence of CHIKV, as described previously^13^. CHIKV infection was confirmed in 1295 (71.7%) of 1806 samples by positive real-time reverse transcriptase–polymerase chain reaction (RT-PCR) results and/or CHIKV IgM antibody, and 511 (28.3%) samples were negative for CHIKV. All samples were re-evaluated to detect ZIKV and DENV by using RT-PCR.

### Detection of ZIKV and DENV by one-step real-time RT-PCR

Total RNA was extracted from 200 μL of each sample using a magLEAD® kit. To screen for ZIKV and DENV, qualitative one-step real-time RT-PCR was performed with specific primer sets and fluorophore-labeled hybridization probes using previously described PCR conditions^59,60^. Amplifications were conducted on an ABI viiA7 Real-Time PCR instrument (Applied Biosystems).

### Determination of DENV serotypes

All DENV-positive samples were subjected to serotype analysis using conventional one-step DENV serotype-specific multiplex RT-PCR according to a previous report^61^, with some modifications. In brief, RNA was used as the template in a total reaction volume of 20 µL of SensiFAST reagent (Bioline, London, UK) with dengue consensus forward primer (D1: 5’-TCAATATGCTGAAACGCGCGAGAAACCG-3’) and four serotype-specific reverse primers for DENV-1 (Ts1: 5’-CGTCTCAGTGATCCGGGGA-3’), DENV-2 (Ts2: 5’-CGCCACAAGGGCCATGAACAG-3’), DENV-3 (Ts3: 5’-TAACATCATCATGAGACAGAGC-3’), and DENV-4 (Ts4: 5’-CTCTGTTGTCTTAAACAAGAGA-3’). The reaction mixture was subjected to the following cycling conditions: cDNA synthesis step at 45 °C for 20 min, initial denaturation at 94 °C for 2 min, followed by 40 replication cycles of denaturation at 94 °C for 30 s, annealing at 55 °C for 45 s, extension at 72 °C for 45 s, and final extension at 72 °C for 5 min. The amplification products were analyzed by 2% agarose gel electrophoresis. Samples positive for DENV-1, DENV-2, DENV-3, or DENV-4 were determined based on detection of a PCR product of 482, 119, 290, or 392 bp, respectively.

### Genotype characterization and phylogenetic analysis

For ZIKV, amplification of the structural protein gene (C-prM-E) and coding-complete genome sequence were performed using a Superscript III one-step RT-PCR system (Thermo Fisher Scientific, Darmstadt, Germany) with previously reported primers and PCR conditions ^62^. For DENV characterization, RNA extracted from serum of confirmed dengue cases with serotype identification was used as the template to amplify the dengue C-prM and E genes. One-step RT-PCR was performed with primers previously designed by Diaz-Badillo et al.^61^ and Cruz et al.^63^ to amplify the C-prM and E genes, respectively. Amplicons were purified using an Expin™ Combo GP kit (General Biosystem, South Korea) according to the manufacturer’s instructions. The purified products were then subjected to Sanger sequencing (1st BASE, Singapore). The resulting nucleotide sequences were analyzed using Chromas Lite version 2.0 and the NCBI Basic Local Alignment Search Tool (BLAST) (http://blast.ncbi.nlm.nih.gov/Blast.cgi). Edited nucleotide sequences were assembled manually using BioEdit Sequence Alignment Editor Software, version 7.0.5.3. All nucleotide sequences were aligned using MUSCLE (multiple sequence comparison by log expectation), implemented in the MEGA 11 program. A maximum-likelihood tree was constructed using the IQ-TREE tool (http://iqtree.cibiv.univie.ac.at/) with optimal model selection and 1000 ultrafast bootstrap replicates. Finally, the constructed phylogenetic tree was visualized and edited using FigTree v.1.4.4 software. All viral gene sequences were deposited in the NCBI GenBank. The ZIKV structural region sequences (C-prM-E) were deposited in GenBank under accession numbers OM936182-OM936195, complete ZIKV coding sequence (accession numbers: OM964565-OM964568), DENV-1 partial E region (accession numbers: ON780003-ON780014), DENV-2 partial E region (accession numbers: ON782110-ON782129), DENV-3 partial prM region (accession numbers: ON799425-ON799432), DENV-4 partial E region (accession numbers: ON787752-ON787756).

### Data analysis

The qualitative data were shown as n (%) and the mean with standard deviation for the day of disease onset detection and age of the patients. The chi-square test and logistic regression analysis were performed to compare demographic variables and the presence of symptoms among different groups of infections. Statistical analysis of significance was analyzed by using SPSS software, version 22. A *p* value of less than 0.05 was considered as statistically significant.

## Supplementary Information

Supplementary Information 1.

Supplementary Information 2.

Supplementary Information 3.

## Data Availability

All data generated during this study are contained within this manuscript and its Supplementary Information files. All sequences generated during the current study are available in the GenBank database, accession numbers OM936182-OM936195 (ZIKV structural region sequences), OM964565-OM964568 (complete ZIKV coding sequence), ON780003-ON780014 (DENV-1 partial E region), ON782110-ON782129 (DENV-2 partial E region), ON799425-ON799432 (DENV-3 partial prM region), ON787752-ON787756 (DENV-4 partial E region).

## References

[1] Paixao ES, Teixeira MG, Rodrigues LC (2018). Zika, chikungunya and dengue: the causes and threats of new and re-emerging arboviral diseases. BMJ Glob. Health.

[2] Campbell KM, Lin CD, Iamsirithaworn S, Scott TW (2013). The complex relationship between weather and dengue virus transmission in Thailand. Am. J. Trop. Med. Hyg..

[3] Bureau of Epidemiology DoDC, M., Thailand. Annual incidence report of dengue virus in Thailand. https://ddc.moph.go.th/dvb/. Accessed 28 July 2021.

[4] Green S, Rothman A (2006). Immunopathological mechanisms in dengue and dengue hemorrhagic fever. Curr. Opin. Infect. Dis..

[5] Holmes EC, Burch SS (2000). The causes and consequences of genetic variation in dengue virus. Trends Microbiol..

[6] Vu TT (2010). Emergence of the Asian 1 genotype of dengue virus serotype 2 in viet nam: In vivo fitness advantage and lineage replacement in South-East Asia. PLoS Negl. Trop. Dis..

[7] Rico-Hesse R (1997). Origins of dengue type 2 viruses associated with increased pathogenicity in the Americas. Virology.

[8] Ruchusatsawat K (2019). Long-term circulation of Zika virus in Thailand: An observational study. Lancet Infect. Dis..

[9] Khongwichit S, Wikan N, Auewarakul P, Smith DR (2018). Zika virus in Thailand. Microbes Infect..

[10] Bureau of Epidemiology DoDC, M., Thailand. Annual incidence report of Zika virus in Thailand. https://ddc.moph.go.th/dvb/. Accessed 20 Apr 2022.

[11] Nimmannitya S, Halstead SB, Cohen SN, Margiotta MR (1969). Dengue and chikungunya virus infection in man in Thailand, 1962–1964. I. Observations on hospitalized patients with hemorrhagic fever. Am. J. Trop. Med. Hyg..

[12] Khongwichit S, Chansaenroj J, Chirathaworn C, Poovorawan Y (2021). Chikungunya virus infection: Molecular biology, clinical characteristics, and epidemiology in Asian countries. J. Biomed. Sci..

[13] Khongwichit S (2021). Large-scale outbreak of Chikungunya virus infection in Thailand, 2018–2019. PLoS ONE.

[14] Agarwal A, Sharma AK, Sukumaran D, Parida M, Dash PK (2016). Two novel epistatic mutations (E1:K211E and E2:V264A) in structural proteins of Chikungunya virus enhance fitness in Aedes aegypti. Virology.

[15] Suwanmanee S (2018). Monitoring arbovirus in Thailand: Surveillance of dengue, chikungunya and zika virus, with a focus on coinfections. Acta Trop..

[16] Liu ZY, Shi WF, Qin CF (2019). The evolution of Zika virus from Asia to the Americas. Nat. Rev. Microbiol..

[17] Luvira V (2019). Etiologies of acute undifferentiated febrile illness in Bangkok, Thailand. Am. J. Trop. Med. Hyg..

[18] Chang C, Ortiz K, Ansari A, Gershwin ME (2016). The Zika outbreak of the 21st century. J. Autoimmun..

[19] Nakkhara P, Chongsuvivatwong V, Thammapalo S (2013). Risk factors for symptomatic and asymptomatic chikungunya infection. Trans. R. Soc. Trop. Med. Hyg..

[20] Ten Bosch QA (2018). Contributions from the silent majority dominate dengue virus transmission. PLoS Pathog..

[21] Silva MMO (2019). Concomitant transmission of dengue, chikungunya, and zika viruses in Brazil: Clinical and epidemiological findings from surveillance for acute febrile illness. Clin. Infect. Dis..

[22] Belaunzaran-Zamudio PF (2021). Comparison of clinical characteristics of Zika and dengue symptomatic infections and other acute illnesses of unidentified origin in Mexico. PLoS Negl. Trop. Dis..

[23] Watzinger F (2004). Real-time quantitative PCR assays for detection and monitoring of pathogenic human viruses in immunosuppressed pediatric patients. J. Clin. Microbiol..

[24] Del Viedma MPM (2019). Optimization of qRT-PCR assay for zika virus detection in human serum and urine. Virus Res..

[25] Guzman MG, Kouri G (2004). Dengue diagnosis, advances and challenges. Int. J. Infect. Dis..

[26] Singh RK (2017). Advances in diagnosis, surveillance, and monitoring of Zika virus: An update. Front. Microbiol..

[27] Zambrano H (2016). Zika virus and chikungunya virus coinfections: A series of three cases from a single center in Ecuador. Am. J. Trop. Med. Hyg..

[28] Prata-Barbosa A (2018). Co-infection with Zika and Chikungunya viruses associated with fetal death-A case report. Int. J. Infect. Dis..

[29] Mercado-Reyes M (2019). Dengue, chikungunya and zika virus coinfection: results of the national surveillance during the zika epidemic in Colombia. Epidemiol. Infect..

[30] Taraphdar D, Sarkar A, Mukhopadhyay BB, Chatterjee S (2012). A comparative study of clinical features between monotypic and dual infection cases with Chikungunya virus and dengue virus in West Bengal, India. Am. J. Trop. Med. Hyg..

[31] Chia PY (2017). Clinical features of patients with Zika and dengue virus co-infection in Singapore. J. Infect..

[32] Lobkowicz L (2020). The frequency and clinical presentation of Zika virus coinfections: A systematic review. BMJ Glob. Health.

[33] Phumee A (2019). Molecular epidemiology and genetic diversity of Zika virus from field-caught mosquitoes in various regions of Thailand. Pathogens.

[34] Yuan L (2017). A single mutation in the prM protein of Zika virus contributes to fetal microcephaly. Science.

[35] Liu Y (2017). Evolutionary enhancement of Zika virus infectivity in Aedes aegypti mosquitoes. Nature.

[36] Pettersson JH (2016). How did Zika virus emerge in the Pacific islands and Latin America?. MBio.

[37] Xia H (2018). An evolutionary NS1 mutation enhances Zika virus evasion of host interferon induction. Nat. Commun..

[38] Wang L (2016). From mosquitos to humans: Genetic evolution of Zika virus. Cell Host Microbe.

[39] Zhao F (2018). Negligible contribution of M2634V substitution to ZIKV pathogenesis in AG6 mice revealed by a bacterial promoter activity reduced infectious clone. Sci. Rep..

[40] Peng NYG (2022). The distinguishing NS5-M114V mutation in American Zika virus isolates has negligible impacts on virus replication and transmission potential. PLoS Negl. Trop. Dis..

[41] Rodrigues LC (2016). Microcephaly and Zika virus infection. Lancet.

[42] Goncalvez AP (2002). Diversity and evolution of the envelope gene of dengue virus type 1. Virology.

[43] Koo C (2018). Highly selective transmission success of dengue virus type 1 lineages in a dynamic virus population: An evolutionary and fitness perspective. iScience.

[44] Shi Y (2016). Epidemiological and molecular characterization of dengue viruses imported into Guangzhou during 2009–2013. Springerplus.

[45] Hamel R (2019). Phylogenetic analysis revealed the co-circulation of four dengue virus serotypes in Southern Thailand. PLoS ONE.

[46] Xu G (2007). An outbreak of dengue virus serotype 1 infection in Cixi, Ningbo, People’s Republic of China, 2004, associated with a traveler from Thailand and high density of Aedes albopictus. Am. J. Trop. Med. Hyg..

[47] Yamashita A, Sasaki T, Kurosu T, Yasunaga T, Ikuta K (2013). Origin and distribution of divergent dengue virus: Novel database construction and phylogenetic analyses. Future Virol..

[48] Mir D, Romero H, de Fagundes Carvalho LM, Bello G (2014). Spatiotemporal dynamics of DENV-2 Asian-American genotype lineages in the Americas. PLoS ONE.

[49] Wang C (2016). Evolutionarily successful Asian 1 dengue virus 2 lineages contain one substitution in envelope that increases sensitivity to polyclonal antibody neutralization. J. Infect. Dis..

[50] Rabaa MA (2013). Frequent in-migration and highly focal transmission of dengue viruses among children in Kamphaeng Phet, Thailand. PLOS Negl. Trop. Dis..

[51] Chang S-F (2016). Laboratory-based surveillance and molecular characterization of dengue viruses in Taiwan, 2014. Am. Soc. Trop. Med. Hyg..

[52] Yenamandra SP (2021). Evolution, heterogeneity and global dispersal of cosmopolitan genotype of Dengue virus type 2. Sci. Rep..

[53] Waman VP, Kale MM, Kulkarni-Kale U (2017). Genetic diversity and evolution of dengue virus serotype 3: A comparative genomics study. Infect. Genet. Evol..

[54] Zhang C (2005). Clade replacements in dengue virus serotypes 1 and 3 are associated with changing serotype prevalence. J. Virol..

[55] Huang J-H (2012). Molecular characterization and phylogenetic analysis of dengue viruses imported into Taiwan during 2008–2010. Am. Soc. Trop. Med. Hyg..

[56] Wittke V (2002). Extinction and rapid emergence of strains of dengue 3 virus during an interepidemic period. Virology.

[57] Phadungsombat J (2018). Emergence of genotype Cosmopolitan of dengue virus type 2 and genotype III of dengue virus type 3 in Thailand. PLoS ONE.

[58] Soe AM (2021). Emergence of a novel dengue virus 3 (DENV-3) genotype-I coincident with increased DENV-3 cases in Yangon, Myanmar between 2017 and 2019. Viruses.

[59] Lanciotti RS (2008). Genetic and serologic properties of Zika virus associated with an epidemic, Yap State, Micronesia, 2007. Emerg. Infect. Dis..

[60] Pongsiri P, Praianantathavorn K, Theamboonlers A, Payungporn S, Poovorawan Y (2012). Multiplex real-time RT-PCR for detecting chikungunya virus and dengue virus. Asian Pac. J. Trop. Med..

[61] Díaz-Badillo A (2014). A DNA microarray-based assay to detect dual infection with two dengue virus serotypes. Sensors.

[62] Leguia M (2017). Full-genome amplification and sequencing of Zika viruses using a targeted amplification approach. J. Virol. Methods.

[63] Cruz CD, Torre A, Troncos G, Lambrechts L, Leguia M (2016). Targeted full-genome amplification and sequencing of dengue virus types 1–4 from South America. J. Virol. Methods.

