# Supplementary material for: Molecular surveillance of arboviruses circulation and co-infection during a large chikungunya virus outbreak in Thailand, October 2018 to February 2020
Source: Sci Rep. 2022 Dec 24;12:22323. doi: 10.1038/s41598-022-27028-7 (PMC9789961; doi:10.1038/s41598-022-27028-7)
Supplement: Supplementary file 1 — Supplementary Information 1. [file 41598_2022_27028_MOESM1_ESM.pdf]

S1 File: Characteristics presented by ZIKV/DENV/CHIKV mono-infection and co-infection cases.

Variable	Number of cases	ZIKV mono-infection n (%)	OR (95% Cl)	DENV mono-infection n (%)	OR (95% Cl)	CHIKV mono-infection n (%)	OR (95% Cl)	CHIKV /DENV co-infection n (%)	OR (95% Cl)	CHIKV /ZIKV co-infection n (%)	OR (95% Cl)	DENV /ZIKV co-infection n (%)	OR (95% Cl)
**Gender**													
Male	788	15 (51.7)	1.4 (0.7-2.9)	19 (52.7)	1.5 (0.8-2.8)	542 (42.2)	0.8 (0.7-1.0)	3 (37.5)	0.8 (0.2-3.3)	2 (66.7)	2.6 (0.2-28.7)	1 (50)	1.3 (0.1-20.7)
Female	1018	14 (48.3)	ref	17 (47.2)	ref	742 (57.8)	ref	5 (62.5)	ref	1 (33.3)	ref	1 (50)	ref
Total	1806	29 (1.6)		36 (2)		1284 (71.1)		8 (0.4)		3 (0.2)		2 (0.1)	
**Age (years) mean ± SD**		36.4 ± 17.5		31.6 ± 12		38.1 ± 16.9		31.4 ± 12.0		53 ± 7		46 ± 15.6	
**Day (s) onset of symptom mean ± SD**		3 ± 1.5		3 ± 1.7		6 ± 11.4		2.3 ± 1.3		3 ± 1.2		3 ± 2.1	
**Symptom**													
Fever	1735	23 (79.3)**	0.1 (0.1-0.4)	36 (100)**	1.5 (0.2-11.1)	1240 (96.6)	1.5 (0.9-2.5)	8 (100)	0.3 (0.0-2.3)	3 (100)	0.1 (0.0-1.2)	2 (50)	0.2 (0.0-2.6)
Rash	804	24 (82.8)**	6.1 (2.3-16.1)	10 (27.8)*	0.5 (0.2-1.0)	645 (50.2)**	2.3 (1.8-2.8)	2 (25)	0.3 (0.1-1.7)	3 (100)	2.9 (0.3-27.8)	2 (50)	1.8 (0.2-21.1)
Arthralgia	1171	15 (51.7)	0.6 (0.3-1.2)	19 (52.8)	0.6 (0.3-1.2)	935 (72.8)**	3.3 (2.7-4.1)	5 (62.5)	0.6 (0.2-2.7)	2 (66.7)	0.8 (0.1-8.5)	-	-
Conjunctivitis	160	5 (17.2)	2.2 (0.8-5.8)	-	-	128 (9.97)	1.7 (1.1-2.5)	-	-	1 (33.3)	4.4 (0.4-49.3	-	-

Chi-square test was used to determine the association between categorical variables among ZIKV mono-infection and none of ZIKV mono-infection cases, DENV mono-infection and none of DENV mono-infection cases, CHIKV mono-infection and none of CHIKV mono-infection cases, CHIKV/DENV co-infection cases with CHIKV and DENV mono-infection cases, CHIKV/ZIKV co-infection case with CHIKV and ZIKV mono-infection cases, DENV/ZIKV co-infection cases with DENV and ZIKV mono-infection cases. * = p value < 0.05 and ** = p value < 0.01. Logistic regression model and the odds ratio (OR) and its 95% confidence interval (Cl) were determined. The mean with standard deviation (SD) was calculated for day of disease onset detection and age of the patients.
