# Supplementary material for: Molecular surveillance of arboviruses circulation and co-infection during a large chikungunya virus outbreak in Thailand, October 2018 to February 2020
Source: Sci Rep. 2022 Dec 24;12:22323. doi: 10.1038/s41598-022-27028-7 (PMC9789961; doi:10.1038/s41598-022-27028-7)
Supplement: Supplementary file 2 — Supplementary Information 2. [file 41598_2022_27028_MOESM2_ESM.pdf]

S2 File: Data for ZIKV-infected patients

ID	Gender	Age	Location (provine)	Date onset of symtomp	Collection date	Day(s) after symptom onset*	ZIKV real-time RT-PCR (ct)
BK1046	Male	12	Phuket	2019-Mar-25	2019-Mar-29	5	36.81
BK1271	Male	42	Samut Prakan	2019-Aug-28	2019-Aug-30	3	34.26
BK1291	Male	53	Bangkok	2019-Aug-29	2019-Sep-04	7	33.51
BK1345	Male	34	Bangkok	2019-Sep-11	2019-Sep-16	6	34.13
BK1350	Male	39	Bangkok	2019-Sep-15	2019-Sep-17	3	30.5
BK1355	Male	46	Chon Buri	2019-Sep-16	2019-Sep-17	2	30.92
BK1359	Female	14	Bangkok	2019-Sep-17	2019-Sep-18	2	31.56
BK1400	Male	52	Bangkok	2019-Sep-24	2019-Sep-25	2	31.32
BK1419	Female	47	Bangkok	2019-Sep-23	2019-Sep-27	5	31.18
BK1463	Female	22	Chon Buri	2019-Oct-01	2019-Oct-03	3	22.7
BK1503	Female	85	Samut Prakan	2019-Oct-07	2019-Oct-10	4	34.34
BK1581	Female	19	Bangkok	2019-Oct-18	2019-Oct-21	4	32.9
BK1716	Female	37	Bangkok	2019-Nov-02	2019-Nov-04	3	31.16
BK1763	Female	35	Samut Sakhon	2019-Nov-06	2019-Nov-06	1	34.96
BK1766	Male	38	Bangkok	2019-Nov-02	2019-Nov-07	6	29.53
BK1812	Male	50	Bangkok	2019-Nov-11	2019-Nov-12	2	37.95
BK1825	Female	55	Bangkok	2019-Nov-10	2019-Nov-12	3	31.06
BK1869	Fenale	22	Samut Sakhon	2019-Nov-14	2019-Nov-17	4	37.68
BK2112	Male	50	Samut Sakhon	2019-Nov-26	2019-Nov-27	2	35.43
BK2217	Female	37	Samut Sakhon	2019-Nov-30	2019-Dec-02	3	32.09
BK2228	Female	1	Samut Prakan	2019-Dec-03	2019-Dec-03	1	34.07
BK2351	Female	61	Bangkok	2019-Dec-09	2019-Dec-12	4	22.04
BK2504	Female	21	Bangkok	2019-Dec-22	2019-Dec-24	3	32.68
BK2570	Male	34	Bangkok	2020-Jan-02	2020-Jan-02	1	31.38
BK2668	Female	31	Bangkok	2020-Jan-10	2020-Jan-12	3	31.04
BK2671	Male	32	Bangkok	2020-Jan-10	2020-Jan-12	3	36.44
BK2671	Male	32	Bangkok	2020-Jan-10	2020-Jan-12	3	32.44
BK2681	Female	37	Samut Sakhon	2020-Jan-10	2020-Jan-12	3	32.23
BK2699	Male	48	Bangkok	2020-Jan-11	2020-Jan-14	4	35.28
BK2709	Female	13	Samut Sakhon	2020-Jan-14	2020-Jan-16	3	32.81
BK2711	Male	66	Samut Sakhon	2020-Jan-15	2020-Jan-16	2	26.84
BK2742	Male	57	Bangkok	2020-Jan-18	2020-Jan-21	4	29.94
BK2763	Male	43	Bangkok	2020-Jan-24	2020-Jan-26	3	30.1
BK2781	Male	43	Bangkok	2020-Jan-28	2020-Jan-28	1	30.86

*Median time from onset of disease to ZIKV detection wes 3 days
