# Supplementary material for: Molecular surveillance of arboviruses circulation and co-infection during a large chikungunya virus outbreak in Thailand, October 2018 to February 2020
Source: Sci Rep. 2022 Dec 24;12:22323. doi: 10.1038/s41598-022-27028-7 (PMC9789961; doi:10.1038/s41598-022-27028-7)
Supplement: Supplementary file 3 — Supplementary Information 3. [file 41598_2022_27028_MOESM3_ESM.pdf]

S3 File: Data for DENV-infected patients

ID	Gender	Age	Location (provine)	Date onset of symtomp	Collection date	Day (s) after symptom onset*	DENV real-time RT-PCR (ct)	Serotype	Genotype
BK1330	Female	26	Bangkok	2019-Sep-04	2019-Sep-12	9	27.04	DENV-1	Genotype I
BK1344	Female	47	Bangkok	2019-Sep-13	2019-Sep-14	2	19.37	DENV-1	Genotype I
BK1469	Female	19	Bangkok	2019-Oct-02	2019-Oct-04	3	22.55	DENV-1	Genotype I
BK1549	Male	52	Bangkok	2019-Oct-13	2019-Oct-18	6	20.27	DENV-1	Genotype I
BK1563	Male	28	Bangkok	2019-Oct-19	2019-Oct-19	1	21.61	DENV-1	Genotype I
BK1702	Male	59	Bangkok	2019-Nov-01	2019-Nov-02	2	19.49	DENV-1	Genotype I
BK2004	Female	28	Bangkok	2019-Nov-22	2019-Nov-23	2	21.4	DENV-1	Genotype I
BK2300	Male	39	Bangkok	2019-Dec-07	2019-Dec-07	1	27.67	DENV-1	Genotype I
BK2544	Male	32	Samut Prakan	2019-Dec-22	2019-Dec-28	3	21.66	DENV-1	Genotype I
BK2582	Male	40	Bangkok	2020-Jan-01	2020-Jan-05	5	27.75	DENV-1	Genotype I
BK2857	Male	28	Bangkok	2020-Feb-15	2020-Feb-17	3	28.17	DENV-1	Genotype I
BK2874	Male	55	Samut Sakhon	2020-Feb-20	2020-Feb-25	6	19.67	DENV-1	Genotype I
BK2882	Female	10	Bangkok	2020-Feb-28	2020-Mar-01	2	22.38	DENV-1	Genotype I
BK1257	Female	47	Bangkok	2019-Aug-26	2019-Aug-27	2	26.14	DENV-2	Asian I
BK1362	Female	25	Chon Buri	2019-Sep-17	2019-Sep-18	2	20.69	DENV-2	Asian I
BK1444	Female	29	Bangkok	2019-Sep-28	2019-Oct-01	4	21.1	DENV-2	Asian I
BK1723	Male	38	Bangkok	2019-Nov-03	2019-Nov-04	2	23.48	DENV-2	Asian I
BK1730	Male	27	Bangkok	2019-Nov-03	2019-Nov-04	2	28.73	DENV-2	Asian I
BK1758	Female	59	Bangkok	2019-Nov-07	2019-Nov-07	1	28.83	DENV-2	Asian I
BK1770	Female	24	Bangkok	2019-Nov-04	2019-Nov-08	5	22.71	DENV-2	Asian I
BK2371	Female	26	Samut Sakhon	2019-Dec-11	2019-Dec-14	4	21.62	DENV-2	Asian I
BK2526	Female	26	Bangkok	2019-Dec-25	2019-Dec-27	3	25.04	DENV-2	Asian I
BK2767	Female	21	Bangkok	2020-Jan-20	2020-Jan-24	5	29.54	DENV-2	Asian I
BK1610	Female	43	Bangkok	2019-Oct-20	2019-Oct-24	5	29.93	DENV-2	Cosmopolitan
BK1824	Female	37	Bangkok	2019-Nov-12	2019-Nov-12	1	23.88	DENV-2	Cosmopolitan
BK1830	Male	9	Bangkok	2019-Nov-10	2019-Nov-11	2	29.61	DENV-2	Cosmopolitan
BK1912	Female	24	Bangkok	2019-Nov-16	2019-Nov-18	3	27.04	DENV-2	Cosmopolitan
BK2189	Male	27	Bangkok	2019-Dec-01	2019-Dec-02	2	26.04	DENV-2	Cosmopolitan
BK2261	Female	40	Bangkok	2019-Dec-04	2019-Dec-04	1	19.97	DENV-2	Cosmopolitan
BK2274	Male	9	Bangkok	2019-Dec-04	2019-Dec-05	2	21.66	DENV-2	Cosmopolitan
BK2414	Female	29	Bangkok	2019-Dec-17	2019-Dec-17	1	26.54	DENV-2	Cosmopolitan
BK2449	Male	24	Bangkok	2019-Dec-19	2019-Dec-20	2	24.58	DENV-2	Cosmopolitan
BK2590	Female	33	Bangkok	2020-Jan-02	2020-Jan-05	4	27.11	DENV-2	Cosmopolitan
BK1667	Male	12	Bangkok	2019-Oct-30	2019-Oct-31	2	32.02	DENV-3	Genotype I
BK2534	Female	45	Bangkok	2019-Dec-25	2019-Dec-29	5	32.02	DENV-3	Genotype I
BK2589	Male	34	Bangkok	2020-Jan-04	2020-Jan-05	2	28.05	DENV-3	Genotype I
BK2623	Female	32	Bangkok	2020-Jan-04	2020-Jan-07	4	18.72	DENV-3	Genotype I
BK2742	Male	57	Bangkok	2020-Jan-18	2020-Jan-21	4	21.48	DENV-3	Genotype I
BK2852	Male	42	Bangkok	2020-Feb-14	2020-Feb-16	3	31.61	DENV-3	Genotype I
BK1963	Male	41	Bangkok	2019-Nov-19	2019-Nov-21	3	31.72	DENV-3	Genotype III
BK2408	Female	36	Bangkok	2019-Dec-14	2019-Dec-16	3	30.27	DENV-3	Genotype III
BK1631	Male	24	Bangkok	2019-Oct-25	2019-Oct-26	2	20.94	DENV-4	Genotype I
BK1763	Female	35	Samut Sakhon	2019-Nov-06	2019-Nov-06	1	28.94	DENV-4	Genotype I
BK2444	Male	26	Bangkok	2019-Dec-18	2019-Dec-19	2	26.67	DENV-4	Genotype I
BK2625	Male	29	Bangkok	2020-Jan-07	2020-Jan-07	1	22.55	DENV-4	Genotype I
BK2630	Male	37	Bangkok	2020-Jan-06	2020-Jan-08	3	27.77	DENV-4	Genotype I

*Median time from onset of disease to DENV detection was 2 days
